# Psychosocial Barriers of Public Transport Use and Social Exclusion among Older Adults: Empirical Evidence from Lahore, Pakistan

**DOI:** 10.3390/ijerph18010185

**Published:** 2020-12-29

**Authors:** Muhammad Ahmad Al-Rashid, Hong Ching Goh, Yong Adilah Shamsul Harumain, Zulfiqar Ali, Tiziana Campisi, Tahir Mahmood

**Affiliations:** 1 Department of Urban and Regional Planning, Faculty of Built Environment, Universiti Malaya, Kuala Lumpur 50603, Malaysia; bva180010@siswa.um.edu.my (M.A.A.-R.); adilah_shamsul@um.edu.my (Y.A.S.H.); 2 Department of City and Regional Planning, School of Architecture and Planning, University of Management and Technology, Lahore 54770, Pakistan; 3 Department of Systems Engineering and Engineering Management, City University of Hong Kong, SAR, China; zulfiqali3@cityu.edu.hk; 4 Faculty of Engineering and Architecture, Kore University of Enna, Cittadella Universitaria, 94100 Enna, Italy; 5 Department of Technology, School of Science and Technology, The Open University of Hong Kong, SAR, China; tmahmood@ouhk.edu.hk

**Keywords:** social exclusion, public transport, psychosocial, older adults

## Abstract

Transport planning and public health have been intertwined historically. The health impact of public transport services, such as social exclusion, is a widely discussed research topic. Social exclusion is a paramount concern for older adults’ health in the wake of emerging global challenges. However, there remains a significant research gap on how psychosocial barriers faced by older adults in using public transport services influence the social exclusion behavior. The present research provides empirical evidence and shows the impact of certain psychosocial barriers of public transportation on older adults’ social exclusion. A total of 243 Pakistani older adults (aged 60–89 years old) voluntarily participated in this cross-sectional study. The participants provided self-reports on their psychosocial barriers (including perceived norms, attitude, personal ability, habits, neighborhood social constraints, and intention) and the corresponding social exclusion. Partial Least Square Structural Equation Modeling (PLS-SEM) was utilized for the data analysis. The structural path model supported the significant associations between psychosocial barriers and social exclusion. Except for perceived descriptive norms, all other psychosocial barriers predicted older adults’ social exclusion. The research portrays the significance of the psychosocial factors to examine social exclusion and offers practical implications for urban and transport planners. The concerned policymakers can use the research findings to develop age-sensitive, socially sustainable, and healthy cities.

## 1. Introduction

Most countries in the world are experiencing a rapidly growing aging population. Recent estimates [1] suggest that the population of older adults aged 65 years and above will rise to 1.4 billion by 2030, from 901 million in 2015. In the rising age, the states should not only be concerned with the economic and policy strains [2] but also with issues of maintaining social support systems, health care programs, and other public utilities for the older adults. Most importantly, the older adults’ long-term care needs, particularly those with disabilities, are of concern that precludes them from participating in everyday activities [3,4]. Several United Nations Agenda 2030 goals aim to promote health and social sustainability in later life [5]. Given the recent global COVID-19 pandemic shock, accomplishing these goals could be constrained, even in the developed world. Thus, awareness of the vulnerability of older adults is essential.

The health and social implications of public transport services, such as social exclusion, are particularly crucial for older adults [3,4,6]. Social exclusion refers to a process in which a person or group cannot participate effectively in mainstream society due to poor accessibility and mobility [7,8]. The process of social exclusion restricts socioeconomic participation, which ultimately affects the health, life quality, cohesion, and equity of the aging society [9,10,11,12]. Older adults in urban environments are more vulnerable to experience marginalization and are likely to be prone to social inequality, comparatively restricted participation in daily activities, and worsening physical conditions [13]. Such a lack of activity participation is deeply rooted in the physical, economic, social, and other mobility constraints [14]. Most importantly, the lack of public transport accessibility could lead to ongoing and increasing social disparities, with the effect of increased social isolation, lowered social wellness, and decreased quality of life [15,16,17]. It reveals that accessibility and mobility are critical issues for research development on social exclusion [18]. Hence, considerable social exclusion challenges require the emergence of radical new ways of thinking about introducing sustainable public transport systems [19].

Social exclusion researchers specifically relate the implications of mobility to the social outcome and bring the dilemma of access to public transport within the framework of a larger social policy agenda [20]. Therefore, the social exclusion paradigm offers tremendous potential for examining the mobility of older adults, as it identifies several factors influencing the access of people to transport [21]. It is also probable that the various facets of mobility affected by the aging process could be considered [22]. In a recent review, Gorman, Jones [23] suggested that social exclusion among older adults has many features that can be defined by their travel experience and have been discussed in the studies that focus on accessibility and transport needs obliquely. Thus, social exclusion features are more substantial than deprivation of income alone and entail the absence of sociocultural and political participation [10]. Implementing a participatory planning strategy can bring to light the critical issues present in an area, considering the population groups initially excluded and evaluating different forms of resolution and mobility implementation [24].

Apart from the narrow biomedical model and physical environmental characteristics, the mobility of older adults is very much dependent on a set of psychosocial factors including the social relationship within the household, their relatives and acquaintances; cultural norms and expectations; attitudes; life-course trajectories; and experiences of individuals and societies [23,25]. The impact of psychosocial influences on the mobility and use of public transportation is a crucial concern for older adults in reducing social exclusions [23]. Similarly, the World Health Organization acknowledged that mobility is affected by the instinctive potential and the surroundings in which older adults reside. In turn, mobility choices are influenced by the built environment, the personal attitude of older adults, and other factors [26].

While older adults have become a significant social policy discussion theme, the psychosocial relationships between later age, expectations, attitudes, and travel behavior are minimally researched [27]. Moreover, recent research does not thoroughly examine the emotional and social attributes of aged people about public transport and how they can be excluded from everyday socioeconomic activities. Hence, the awareness of the psychosocial features of public transport users is vital for a better understanding of social exclusion among older adults [28,29].

The psychosocial factors have been extensively recognized in the existing research to predict public transport use, mobility, and travel behavior [30,31,32,33,34,35,36]. Most importantly, these factors show substantial implications among older adults [32,33,34,35,36]. However, there is a limited understanding of how the psychosocial characteristics of public transit users influence social exclusion behavior. The Integrated Behavioral Model (IBM) offers an established framework of the psychosocial factors to examine the individual’s behavior [37]. Therefore, the present research utilized the IBM framework and identified the critical psychosocial aspects in predicting social exclusion.

The theoretical background and suggested hypotheses for this research are presented in Section 2; study context, data collection, and methodology are explained in Section 3; study results are briefly discussed and interpreted in Section 4 and Section 5; while later sections conclude this paper and provide practical implications, limitations, and suggestions.

## 2. Theoretical Background

The Integrated Behavioral Model (IBM) was developed by Martin Fishbein and his colleagues from several well-established theories, including the Theory of Reasoned Action (TRA), Theory of Planned Behavior (TPB), and Social Cognitive Theory [37]. The model describes behavioral intentions as the most influential factor in performing a behavior. Aside from intentions, personal skills, expertise, understandings, and environmental restrictions (both social and physical) directly impact the behavior [38]. It implies that one must be competent to carry out actions, and it is probable that behavior will not be performed when there are environmental restrictions. Intentions can be further predicted by three core constructs, including behavioral attitudes (overall personal evaluation of a behavior), personal agency (reflecting the belief of a person that he or she possess an autonomy and able to conduct a behavior), and perceived norms (a person’s understandings of social pressure to enact a behavior) [37,39].

The TRA and TPB constructs of subjective norms include normative assumptions (i.e., an individual’s presumption of social pressure of adopting the behavior) and an encouragement to conform [40]. Even though TRA and TPB support the notion of subjective norms, the theoretical design of IBM includes both injunctive and descriptive norms. IBM may be a more comprehensive framework to predict public transport use than TRA as it integrates self-efficacy and perceived behavioral control within the personal agency construct. While TPB includes the idea of perceived behavioral control, IBM may also be more substantial than TPB because IBM encompasses other theoretically significant constructs to public transport and social exclusion research such as environmental constraints and personal habits.

**Figure 1:**
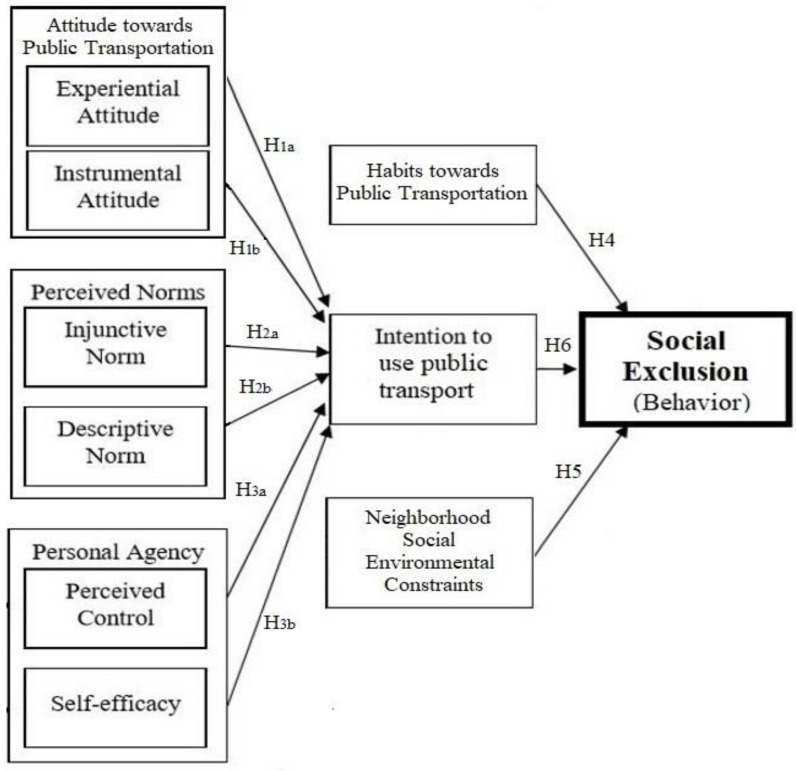
Conceptual framework of the study adapted from the Integrated Behavioral Model [37].

Theory-based determinants of social exclusion have not been sufficiently applied or studied in public transportation research. Given the intrinsic gains of theoretical approaches, efforts need to be made to use behavioral theories when designing and planning the public transport interventions, such as identifying the socio-psychological influences of transport users and specifying methods to manage social exclusion behaviors. While many travel behavior models and theories exist in transportation research [31,41,42], the Integrative Behavioral Model (IBM) is an emerging theory in the field and warrants further investigation. IBM has general benefits over other theories in behavioral research. For instance, IBM blends leading behavioral and social science research theories with the most useful constructs and offers a clear foundation for using the whole model or constructs from the model where possible [38]. Thus, to the best of the authors’ knowledge, no published research has utilized IBM as a framework for social exclusion research. Therefore, given the comprehensiveness and versatility of the model, IBM constructs have been selected as part of the conceptual framework for the present study. Figure 1 shows the proposed conceptual framework and research hypotheses.

### 2.1. Development of Hypotheses

#### 2.1.1. The Effects of Attitude on the Intention to Use Public Transportation

The attitude reflects a personal assessment of the perceived advantages and disadvantages of performing a given behavior [43]. Attitudes may be described as instrumental (cognitive) or experiential (affective). Instrumental attitude focuses on the benefit and function characters [44], whereas experiential attitudes are emotional responses generated by thinking about behavior and are based on emotional aspects and feelings about the behavior [37]. The acceptability of each system has been mainly decided by attitudes [45]. The association between intention and attitude, as explained by [31] in the Theory of Planned Behavior, is a theoretical context or a reference point to construct a more theoretical viewpoint with respect to subjects. Hence, it is evident that attitudes to agree or disagree with something are very important [45].

In the formation of public transport-related attitudes, the social component is relevant. According to [46], detrimental social expectations and perceived unfairness may be possible considerations in determining the acceptability of public transportation. The cultures in some countries have a negative attitude or unfavorable impact on public transport use, especially among disadvantaged population groups such as women [47,48]. Hence, studying the attitudes and conditions of public transport usage has gained prime importance rather than evaluating the physical proximity measures [49].

It is well established that attitudes toward certain travel modes significantly influence the behavior with the mediating effect of intentions [31,50,51]. Bopp, Kaczynski [52] exposed that people with the highest ranks of eco-friendly attitudes possessed a greater probability of active commuting than the people with lesser rankings. The study further found that the people of the top ecological quartiles also achieved greater self-efficacy and had fewer barriers to active travel. Similarly, in tourism research, [53] provided an insight into the relationship between voluntary tourists’ attitudes and their intended involvement. Additionally, a recent study [54] suggested that a positive attitude among older adults toward a public transport mode could lead to more social interaction and increased community involvement.

Therefore, studying older adults’ attitudes is particularly relevant because of their growing dependence on private cars, political and demographic significance, and their susceptibility to transport-related social exclusion [28]. However, to the best of our knowledge, there is no such study investigating the direct association between the attitude of the older adults and the intention to use public transportation. Thus, it is hypothesized that:

**Hypothesis** **1** **(H1).** *There is an association between attitude and intention to use public transportation among older adults.*

**Hypothesis** **1a** **(H1a).** *Experiential attitude is positively associated with the intention to use public transportation among older adults.*

**Hypothesis** **1b** **(H1b).** *Instrumental attitude is positively associated with the intention to use public transportation among older adults.*

#### 2.1.2. The Effects of Perceived Norms on the Intention to use Public Transportation

Perceived norms, or in other words, social norms, are usually seen as social precedents connected to collective group characteristics but may also be interpreted individually [55]. As defined by Ajzen [56], the social norm reflects an individual’s perception of social pressures to carry out a particular action or not. It is possible to view perceived social norms as descriptive and injunctive norms. Cialdini [57] addressed descriptive norms as what is usually done within a group of people. In contrast, injunctive norms impose on a group of people whether a behavior is traditionally accepted or rejected.

In the context of transportation, the existing research primarily explains how social norms influence travelers’ attitudes and intentions, travel behavior and pattern, mode use, and choice decision [47,58,59,60,61,62,63,64]. The research supports the interpretation of an individuals’ higher subjective descriptive and injunctive norm, the more likely they are to perform a particular behavior [65]. Moreover, it is also accepted that perceived norms significantly influence intention [66], which is an immediate antecedent of action to choose public transport mode. In other words, if a person begins to understand that essential referents (such as family members, colleagues, close friends) agree that travel should be environmentally friendly, he may feel less pressure and plan to use active travel and public transport [62,67,68]. Therefore, it can be assumed that a better perception of social norms would affect the intention to use public transportation. To the best of the authors’ knowledge, no such study proves the direct associations between perceived norms and intention to public transport use among older adults. For a better understanding of these research gaps, we have proposed the hypotheses that state that:

**Hypothesis** **2** **(H2).** *There is an association between perceived norms and intention to use public transportation among older adults.*

**Hypothesis** **2a** **(H2a).** *Injunctive norms are positively associated with the intention to use public transportation among older adults.*

**Hypothesis** **2b** **(H2b).** *Descriptive norms are positively associated with the intention to use public transportation among older adults.*

#### 2.1.3. The Effects of Personal Agency on the Intention to Use Public Transportation

The personal agency comprises the control beliefs and efficacy beliefs [37]. There is an argument that self-efficacy differs from perceived behavioral control (PBC). The former suggests that people view their actions as controlled by their will. It includes internal and external attributes, such as personal ability, confidence, mood, time, income, etc. [69]. Simultaneously, the latter indicates the extent to which a person perceives the ease or difficulty of performing a particular activity [69,70]. In addition, [71] claimed that self-efficacy could better predict the individuals’ evaluation of abilities than perceived behavioral control. Thus, both aspects of personal agency are incredibly essential to analyze an individual’s capabilities toward a specific activity.

Studies [72,73] enlightened that when an individual has a high degree of self-efficacy, he welcomes a challenge, feeling highly competent and confident in coping with his ability to navigate change. Along the same lines, [74] also suggested self-efficacy as a vital factor that could enhance volunteer tourists’ participation because an individual must have a certain degree of skills and abilities in performing volunteer work over unforeseen obstacles. Furthermore, self-efficacy is strengthened once the individual participates in voluntary activities. Perceived behavioral control and environmental expectations may also have beneficial implications for physical activity [75]. In this regard, [43] found that attitude and perceived behavioral control directly affect physical activity intentions. In another study, physical activity was projected by motivational factors, including perceived behavioral control and intentions [76]. Hence, it can be said that the more people feel they possess the skills, necessary resources, abilities, and opportunities required to affect behavior, the more likely they would intend and continue to act [77]. In line with these arguments, we proposed the following hypotheses.

**Hypothesis** **3** **(H3).** *There is an association between personal agency and intention to use public transportation among older adults.*

**Hypothesis** **3a** **(H3a).** *Perceived behavioral control is positively associated with the intention to use public transportation among older adults.*

**Hypothesis** **3b** **(H3b).** *Self-efficacy is positively associated with the intention to use public transportation among older adults.*

#### 2.1.4. The Effects of Public Transport Habits on Social Exclusion

Habits are automatic behavioral responses, where people tend to repeat the same actions in recurring contexts [78]. Habits play an important role in influencing one’s actions and are commonly taken into account to interpret and predict human behavior [79]. The use of a particular travel mode might be seen as a habitual behavior, as it can be routinely performed without conscious consideration [50]. Psychological research has also revealed that past behaviors are a good predictor of future actions [80]. Earlier work has indicated that people who possess a strong habit toward a specific mode of transport (e.g., private cars) obtained fewer details and focused less on other transportation choices [81]. Moreover, several scholars have also suggested that motives, attitudes, and intentions lose influence when using a specific transport mode becomes a habit [82,83].

The associations between habitual factors, mode use, and travel behavior are consistently found in the existing literature. For instance, car use habits possess negative relationships with public transport use but are positively related to car use [81,82]. Similarly, Şimşekoğlu, Nordfjærn [50] revealed the car use habit as a negative indicator of public transport use. Moreover, Beltran, Wilson [84] found that people with strong active transport habits frequently carry out active travel to their destinations and have a lower Body Mass Index (BMI). People who engage habitually in their favorite activities realize past success and predictable consequences and will likely be involved in similar activities [85]. It might be argued that the individuals who are habitual of using public transportation will continue to avail these facilities. Such increased use of public transit will promote independent mobility among older adults [86,87,88]. Thus, in turn, it is helpful in reducing social exclusions. Consistent with these theoretical arguments, the present research posits that strong habits toward using public transportation lead to reduced social exclusion. Accordingly, the following hypothesis is suggested:

**Hypothesis** **4** **(H4).** *Habits of using public transportation are negatively related to the social exclusions among older adults.*

#### 2.1.5. The Effects of Neighborhood Social Constraints of Public Transportation on Social Exclusion

A neighborhood is one of the central locations we experience frequently and has social and physical characteristics [89,90]. Fornara, Bonaiuto [91] revealed three crucial aspects of the social environment: neighborhood security, discretion, and sociability. Existing transportation research richly emphasized the importance of neighborhood social environment. It does seem to affect physical activity, transport walking, mobility, travel intentions, and behavior [32,33,89,92,93,94]. Several neighborhood social factors tend to be associated as far as physical activity is concerned. For example, Kaczynski and Glover [92] discovered that people are more involved in physical activity if people see their surroundings as pedestrian-friendly and socially connected. There is also a malicious link between crime and lack of physical activity [93,95,96]. Likewise, physical activity, the neighborhood’s social features also affect walking behavior and intention [89]. Moreover, social features also affect the use of public transportation [97].

While significant transport and psychological researchers have examined neighborhood social environment, little empirical data have shown the potential relationship between neighborhood social constraints and social exclusions. This research establishes a link between neighborhood social constraints of public transportation and social exclusion behavior to address this literature gap. According to previous research [97], a person perceiving more significant social limitations in their community is more likely to experience restrictions in civic and political activities; hence, it is fair to conclude a positive relationship. Thus, the following hypothesis is submitted:

**Hypothesis** **5** **(H5).** *Perceiving neighborhood social environment constraints while using public transportation is positively related to older adults’ social exclusion.*

#### 2.1.6. The Effects of Intention to Use Public Transportation on Social Exclusion

The importance of intention is keenly discussed in numerous behavioral theories such as Theory of Planned Behavior, Theory of Reasoned Action, and Theory of Interpersonal Behavior [40,98]. The personal desire to perform a particular activity is the most proximal determinant of the behavior [98]. Several social psychological research studies have investigated behavioral intentions in response to social exclusion [99,100,101].

For example, Bacon [101] examined the interrelationships among social exclusion and harmful behavior and confirmed the increased use of alcohol due to social rejections. However, limited research has focused on the direct impact of intention on social exclusion. In one of the few studies on this subject, Sano and Kyougoku [102] suggested that achievement motivation (i.e., intentions to achieve one’s goal) is positively correlated with social participation.

Moreover, Alnahdi and Schwab [103], in their research on students with intellectual disabilities, found that behavioral intention to interact with peers promotes social participation and can help reduce social exclusion. No study has investigated how the intention to use public transportation is associated with social exclusion. Based on the previous literature findings, a person with greater intention to adopt a certain behavior promotes social participation [102,103], thus reducing social exclusion. Hence, the following hypothesis is suggested:

**Hypothesis** **6** **(H6).** *Intention to use public transportation is negatively associated with social exclusion among older adults.*

## 3. Methodology

### 3.1. Study Context and Data Collection

**Figure 2:**
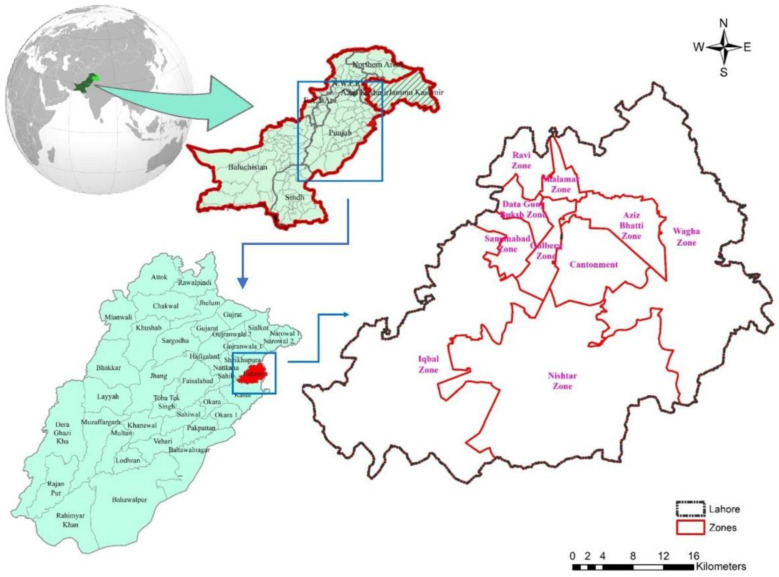
Zone-level administrative map of the city district of Lahore, adapted from [104].

The research was carried out in Lahore city, which is one of the districts in Punjab province of Pakistan. It is situated in the northeast of Punjab close to the Indian border and extends from 74°10′ and 74°39′ E longitude and 31°15′ and 31°43′ N latitude. The Lahore City District comprises of one cantonment and nine administrative zones (Figure 2). These zones are further sub-isolated into 150 Union Councils (UCs) or neighborhoods [104]. Lahore holds a diversity of urban public transport services that can be categorized into three types: (1) green and orange line metro, (2) intra-city bus services; and (3) paratransit services [105]. The public bus and metro services are mostly operated and owned by the public sector. Punjab Mass-transit Authority (PMA) regulates the metro services, and Lahore Transport Company (LTC) manages the intra-city bus services. PMA and LTC have taken various fiscal measures to attract older adults toward these public transport services. For instance, the “Free Transport Card” allows senior citizens (above 60 years) and disabled persons to travel free of cost on LTC Buses within Lahore city [106]. Similarly, PMA also offers discount ticket worth Rs. 30 (1 PKR = 0.0062 US $) for a complete one-way journey [107]. However, there is no subsidy on the paratransit services that are mostly owned and operated by the private sector.

The metro users can either buy the ticket or token from the on-site ticket booth at the station or from any of the self-service Ticket Vending Machines (TVM) available [108]. The frequent travelers can also get a Metrobus card with a safety deposit of around Rs. 130. These credit card-sized cards make it extremely convenient to travel on the metro services, as users do not need to wait in line to buy a ticket for every journey. The card can be recharged at any TVM or a ticket booth if the balance gets used up [109]. The LTC bus users can only buy tokens during the ride within the buses, or they can show their discount card to avoid fare. However, there are no such arrangements for pre-online payments [109]. Regardless of the financial incentives provided by the public transport authorities to the older adults, their knowledge of getting a “token” from the vending machines or use of a smart card to access the metro services is limited, especially among less educated adults [110]. Moreover, older adults also experience numerous psychological barriers in accessing and using the public transport services [105,110,111].

The cross-sectional data were collected via a face-to-face close-ended questionnaire survey from the older adults aged 60 years and above and residing in selected neighborhoods of Lahore, Pakistan. A multi-stage sampling approach was adopted to recruit the participants, where two out of nine Municipal Administrative Zones were selected. Among these chosen zones, a survey via simple random sampling was done in six selected neighborhoods. The criteria for the selection of administrative zones and the corresponding neighborhoods are:

i. Present within 800-m catchment area of green line Metrobus service, Lahore
ii. Possess a greater proportion of older adults, i.e., the more significant aged population as compared to other neighborhoods
iii. Variety of neighborhood and socioeconomic characteristics (high and low-income neighborhoods)

The sample size was computed with G-power software. Memon, Ting [112] highlighted the importance of power analysis using the G-Power program. The minimum sample size calculated by G-power for this research was 189, with an α probability error of 0.05. The sample size calculated by G-power is at a minimum level. Considering these recommendations, a total of 310 older adults were approached. The response rate was 85%. After sorting the incomplete questionnaires, a sample of 243 older adults was considered in this study.

### 3.2. Measure Development

The questionnaire began with a brief explanation of participants’ demographic and socioeconomic information followed by general travel information. The other sections included the questions or items that evaluated the following variables: personal attitude, perceived norms, personal agency (perceived behavioral control and self-efficacy), habits, neighborhood social environment constraints, intentions, and social exclusion.

Attitude. The attitude toward public transportation was categorized into two types: (a) experiential and (b) instrumental attitude. The scale was adapted from [113,114]. Four items measured the experiential attitude, while three items represented the instrumental attitude of older adults. All these items were measured on a five-point Likert scale that ranges from 1—strongly disagree to 5—strongly agree. The greater score represents a positive attitude toward public transport use and vice versa.

Perceived norms. One of the independent variables used in this study is the perceived norm. Perceived norms of public transport were measured by adapting the items previously established by Forward [115]. Perceived descriptive norms were assessed via three measurement items about public transport by the participant’s friends, family, and colleagues. Similarly, a three-item scale was used for injunctive norms in which participants were asked if people they know would allow them to use public transportation. The five-point Likert scale assessed these 6 items, i.e., from 1—strongly disagree to 5—strongly agree). The higher score indicates more significant perceived norms toward public transport usage and vice versa.

Personal Agency. As discussed earlier, perceived behavioral control and self-efficacy were considered as a construct for personal agency. Three items based on the work of [116] were adopted to assess the perceived behavioral control. At the same time, self-efficacy was assessed by the 13-item scale adapted from [117]. All the scale items were assessed on a 5-point Likert scale varying from 1—strongly disagree to 5—strongly agree. The selected scales revealed significant reliability across various population groups and transport modes.

Habit. The strength of public transport habits was recorded using three items developed by [118]. Similar to other scales, the items were judged by a 5-point Likert scale (1—strongly disagree to 5—strongly agree). The higher the score, the more public transport-related habits the respondent has.

Neighborhood social environment. In this study, the neighborhood social environment constraint was selected as one of the independent variables. These perceptions of these constraints were identified by the shorter version of some designated scales assessing Perceived Residential Environment Quality Indicators (PRE-QIs) [91]. Given the scope of this study, only social–relational aspects were chosen from the PRE-QIs scale. The 9-item scale covering the social environment constraints of the neighborhood includes sociability, security, and discretion. A five-point Likert scale assessed the statements. The lower score indicates the low perception of the constraints and vice versa.

Intention. The two-item scale identifying the public transport use intention was adapted from [119]. The 5-point Likert scale (1—strongly disagree to 5—strongly agree) judged older adults’ level of intention. The greater the score, the higher the intention of older adults toward the use of public transportation.

Social exclusion. The dependent variable was social exclusion, which was measured using a scale constructed by Currie and Delbosc [120]. The 5-item scale covers five social exclusion dimensions, covering income, unemployment, political engagement, participation, and social support. Older adults were classified into four different categories to assess the gross-household income level. One item assessed the unemployment status of older adults that included unemployed people due to illness or any disability and those looking for a job. Political engagement was measured on a 5-point Likert scale by recoding the recent participation in community or political groups (1—daily to 5—never). Similarly, older adults were asked if they were excluded from different activities, including sports, leisure, and hobbies, on a 5-point Likert scale (1—strongly disagree to 5—strongly agree). Moreover, social support was also assessed on a 5-point Likert scale by asking how easily they could get support from others when necessary (1—very easy to 5—very difficult).

### 3.3. Data Analysis

The descriptive statistics for the respondents and critical variables used were reported with the IBM Statistical Package for the Social Sciences (SPSS) version 21 (IBM, Armonk, NY, USA). This study employed a partial least squares structural equation modeling (PLS-SEM) method. More specifically, SmartPLS3 (GmbH, Bönningstedt, Germany) [121] was used to assess the research model. This study followed a two-step approach recommended by [122,123] to analyze and interpret the PLS-SEM results: (1) assessment of reliability and validity of the measurement model (or outer model) and (2) testing of the structural model (or inner model).

The following points summarize why this research considers PLS-SEM as a more appropriate statistical technique: (1) the structural model is complex and encompass a series of dependence relationships; (2) the research objective of the structural model is prediction oriented and explains the variance in key target constructs; (3) this study analyzes the relationships between the attitudes, perceived norms, and personal agency as the antecedents of the intention of public transport use. In addition, the influence of neighborhood social constraints, habits, and intention toward public transportation on the dependent variable, i.e., social exclusion, was examined; (4) the sample size (*n* = 243) is also assumed to be relatively small; lastly, (5) this study adopts the advantage of PLS-SEM in terms of less rigorous requirement of restrictive assumption as it enables researchers to create and estimate such models without imposing additional limiting constraints [124].

## 4. Results

### 4.1. Sample Characteristics

The descriptive results show that the majority (52%) of the participants were females, and 48% were males. Most of the older adults were aged 60–64 years, while only 10% of the respondents were 75 years and above. Older adults were selected randomly from each union council, and statistics showed a balanced representation of the participants. Family types of respondents indicated that 30% of older adults belonged to the nuclear family system, while 70% were part of the joint family. Results also showed that a higher number of respondents belonged to a joint or extended family living with their children. However, only a minimal proportion is living alone or with their life partners.

Outcomes of older adults’ employment status indicated that around 50% could not work or were unemployed mainly due to age. Similarly, 38% were retired and choose not to work. However, there is a limited proportion involved in full-time or part-time employment.

Of the participants, 20% of the respondents were illiterate, 40% of older adults had high school qualifications, 24% of older adults were graduates, and only 14% of respondents were qualified up to the post-graduate level. In view of the findings, it is concluded that the level of education of the higher number of older adults was below the undergraduate degree. Thus, the findings of this study would benefit older adults who have a low educational level, possess poor knowledge about automatic fare collection system of public transport services, and are likely to be more socially excluded.

The monthly income of 36% of the participants was below PKR 25,000; 16% of participants had a monthly income between PKR 25,000 and 49,999; 32% of participants responded that their monthly income was between PKR 50,000 and 74,999; and 16% of participants had a monthly income above PKR 74,000. Statistics indicated that the monthly income of a more significant number of participants was below PKR 50,000, which is considered the middle class in the social status ranking. Such respondents were not able to enjoy traveling luxuries owing to their financial barriers.

### 4.2. Model Assessment and Hypotheses Testing

#### 4.2.1. Assessment of the Measurement Model

When the measurement model employs PLS-SEM, the assessment of the individual reliability of the reflective items depends on examining the factor loadings or simple correlations of the reflective dimensions with their respective construct. In this study, the validity and reliability of all the reflective first-order items were assessed to confirm the uni-dimensionality of the constructs. Table 1 lists all the reflective items loadings of the constructs. Generally, all reflective items’ loadings were found to be above 0.70 and were significant at the 0.001 level except for one item, which was dropped in the final analyses to guarantee the convergent validity of the scales.

The measure for convergent validity and construct reliability depicts measures of internal consistency reliability and validity for reflective items. Convergent validity was evaluated by examining the average variance extracted (AVE), which provides the amount of variance that a construct obtains from its items in relation to the amount of the variance due to the measurement error [125]. Table 1 show that the values of AVE of all the constructs are greater than 0.50 at the construct level. Therefore, the convergent validity of the measurement model was acceptable.

The measures of construct reliability include composite reliability and Cronbach’s alpha. This study detailed both composite reliability and Cronbach’s alpha because [123] suggested that researchers should examine composite reliability, Cronbach’s alpha, and AVE to assess reflective construct properties. Table 1 shows that all values of composite reliability and Cronbach’s alpha are greater than or equal to 0.70, suggesting adequate reliability.

This study used Fornell–Larcker criterion and the Heterotrait–Monotrait (HTMT) ratio [125,126] to measure discriminant validity. Fornell–Lacker criterion was assessed by comparing the values of square root of AVE and correlations between the focal construct and all other constructs [125]. Table 2 revealed that all the diagonal values are greater than the correlations of other constructs. Likewise, the second criterion was to measure the validity of the constructs, including the two commonly used parameters with the cutoff points HTMT.85 and HTMT.90, respectively, to evaluate the HTMT values. The values shown in Table 2 are less than the threshold values [121].

#### 4.2.2. Assessment of the Structural Model

This study draws the results of the structural model on Hair et al. [124] by looking at the R^2^, beta (β), and corresponding t-values via bootstrapping. The variance inflation factor (VIF) was assessed, and it was found that the collinearity among the predictor constructs was not an issue in the structural model, as all VIF values were below the threshold of 5. Next, we looked at the relationships between the variables by examining beta values (β) of path coefficients and t-values [123,124]. All the hypotheses were supported except for H2(b). Table 3 shows the results.

Moreover, experiential attitude, instrumental attitude, injunctive norm, descriptive norms, perceived behavioral control, and self-efficacy explained 44% of variance in intentions to use public transport, and intentions to use public transport explained 47% of variance in social exclusion. The R^2^ values of 0.44 and 0.47 were quite acceptable and indicated a substantial model [127]. Model validation was done by standardized root means square residual (SRMR). Analysis produced a value of 0.04 for SRMR, which established the model validation [128].

H1 stated that there would be an association between attitude and intention to use public transportation among older adults. Results suggested that there was a significant positive association between experiential attitude and public transport use intention (*β* = 0.569, *t* = 4.166, *p* = 0.001). Furthermore, instrumental attitude was also found to be a substantial positive determinant of public transport use intentions (*β* = 0.451, *t* = 2.873, *p* = 0.004). Hence, the H1 was fully accepted. It could be revealed that the attitudes on experiential and instrumental attributes of public transportation have a positive influence on the use of public transportation among older adults. In other words, the improvement in instrumental and experiential dimensions could enhance the use of public transport.

Similar results about H2 were reported, which anticipated that perceived norms would positively impact the public transport use intention. The hypothesis results showed a positive and statistically significant influence of injunctive norms on the intention (*β* = 0.601, *t* = 8.088, *p* = 0.001). It revealed that older adults who received acceptance from their peers to use public transportation possess a greater intention to use this mode. On the contrary, descriptive norms did not prove to be a substantial and positive determinant of intention to use public transportation (*β* = 0.051, *t* = 1.345, *p* = 0.321). It disclosed that older adults’ intentions might not be influenced regardless of the frequent use of public transportation among their family members, friends, and peers. Thus, H2 was partially accepted.

Moreover, H3 proposed that there would be a positive association between personal agency and intention to use public transportation. Both H3a and H3b were accepted because perceived behavioral control turned out to be positive significant predictor (*β* = 0.121, *t* = 2.202, *p* = 0.038) and self-efficacy also positively predicted intention to use public transportation (*β* = 0.317, *t* = 2.673, *p* = 0.008). It can be said that older adults who perceive ease in using public transportation could have greater intentions. Similarly, the greater personal ability and self-confidence of older adults in accessing public transportation might instigate its usage.

Considering the direct associations with social exclusion behavior, H4 proposed that public transportation habits are negatively related to older adults’ social exclusions. The summary of the results of the hypotheses (Table 3) revealed a statistically significant negative association between habits and social exclusion (*β* = −0.193, *t* = 3.205, *p* = 0.004). Therefore, H4 was accepted. The finding revealed that using public transportation might improve the daily mobility of older adults, and they could independently participate in sociocultural activities resulting in a reduction of social exclusion.

H5 predicted that perceiving neighborhood social environment constraints would positively affect social exclusion behavior. The results (*β* = 0.496, *t* = 6.203, *p* = 0.000) revealed that the neighborhood social environment constraints are the significant predictors of social exclusion. Therefore, H5 was accepted. It exposed that the perception of neighborhood social constraints is an important aspect of independent mobility and accessibility with public transportation, which might exaggerate social exclusion.

**Figure 3:**
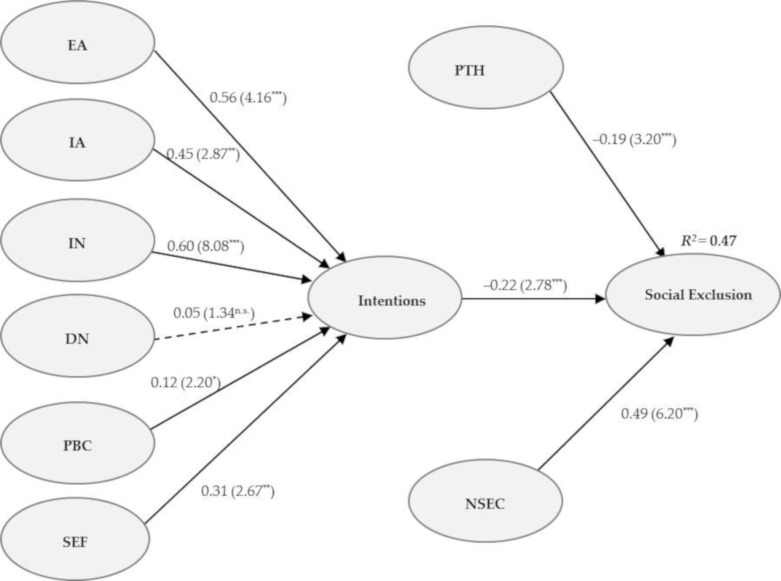
Model estimation results. Figure notes: EA = experiential attitude; IA = instrumental attitude; IN = injunctive norm; DN = descriptive norm; PBC = perceived behavioral control; SEF = self-efficacy; PTH = public transport habits; NSEC = neighborhood social environment constraints. n.s. = Not significant; * significant with |*t*| ≥ 1.65 at *p* 0.05 level; ** significant with |*t*| ≥ 2.33 at *p* 0.01 level; *** significant with |*t*| ≥ 3.09 at *p* 0.001 level; *R^2^* = Determination coefficients

Lastly, the intention to use public transportation was hypothesized to have a negative impact on social exclusion. Indeed, it was found to be a significant negative determinant of social exclusion (*β* = −0.226, *t* = 2.777, *p* = 0.006). Hence, H6 was also accepted. It derived that the older adults who possess greater intentions to use public transportation might travel more frequently and easily participate in their daily activities and thus experience lesser social exclusion. The model estimation results are shown in Figure 3.

In summary, the findings suggested that personal agency had a weaker association with public transport use intention than the perceived injunctive norms and attitudes. Except for perceived descriptive norms, the relationship between all consequence and antecedent variables was supported by our empirical evidence. Thus, all these findings showed that the Integrated Behavioral Model (IBM) could explain public transport-related social exclusion among older adults.

## 5. Discussion

The rapidly growing older population worldwide is seen as a significant socioeconomic challenge, especially in urban areas. Most importantly, the social implications of public transportation, such as social exclusion issues, have attracted the attention of policymakers and researchers during recent times [3]. Until now, social exclusion researchers primarily focused on deriving the measures of transport disadvantages based on the physical and spatial aspects of public transport accessibility [8]. On one hand, the existing literature has abundantly addressed the specific determinants of older people’s mobility and accessibility, including psychological characteristics [29], situational [35], and sociological factors [32,36]. However, few studies have looked at how psychosocial factors of public transport users influence social exclusion behavior. Theoretically grounded in the Integrated Behavioral Model [37], the present study examines the importance of older adults’ psychosocial characteristics to expand the current empirical and theoretical knowledge in the social exclusion domain.

The results disclosed that both aspects of attitude and personal agency directly affected public transport use intentions. The study results partially confirmed the direct associations between perceived norms and intentions. This research also sheds light on the critical roles of public transport habits and neighborhood social environment constraints. The results demonstrated that the perceptions of neighborhood social constraints significantly positively influenced the social exclusion behavior of older adults. In contrast, the direct effect of personal habits on social exclusion was found to be negatively significant. Lastly and most importantly, the intention to use public transportation was a significant negative predictor of social exclusion. These findings contribute significantly to explaining the social exclusion behavior of older adults in Pakistan. This empirical research is one of the first to examine the social exclusion behavior and surveyed public transport users in the selected neighborhoods of Lahore, Pakistan.

Interestingly, as hypothesized, the association between attitudes and public transport use intentions established a positive correlation. Indeed, it emerges that the more critical older adults possess positive attitudes toward public transport, the more likely they would perceive using this mode as attractive and intriguing. Comparing the co-efficient among experiential and instrumental attitudes shows that experiential attitudes are the most influential determinant of intention to use public transportation. Even though the instrumental attributes are considered essential, it reflects that the experiences with public transportation, such as service quality, sensitivity, usefulness, and effectiveness, more significantly shape the attitudes and eventually influence older adults’ intentions.

The results illustrated a significant positive effect of injunctive norms and an insignificant effect of descriptive norms for the association between perceived norms and intention toward public transport use. These results are in line with [67], which suggested that if a person perceives a high degree of social pressure from their peers, the green travel intention will enhance. The results signify the importance of injunctive norms of public transport use in Pakistan, i.e., the older adults could use public transport modes if they receive permission from their peers. The result also reflects the robust family system, and cultural values persist in the society where older adults are considered important and respectable for their children or caretakers. Moreover, children and caretakers also prefer to accompany their older adults for daily travel necessities. So, older adults in Pakistan remain dependent on their peers for their everyday mobility.

The current study established a significant relationship between personal agency and intention to use public transportation. The results revealed that self-efficacy and perceived behavioral control were positively linked with the intention of public transport use. These results supported the findings of previous scholarships [43,74] that confirmed the importance of efficacy and behavioral control on the physical activity intentions. It is plausible that ease of use and personal ability are essential aspects to influence public transport use. Thus, the absence of age-friendly infrastructure could reduce the self-confidence and ability of older adults, which negatively affects the personal intentions to use public transportation.

In this study, strong public transport habits were found to be related to social exclusion. It revealed that older adults who habitually use public transportation might participate in their preferred activities quite often. This outcome corroborates [84], who suggested that people holding active travel habits carry out active travel more frequently and are more physically active. The habits of using public transportation could promote the independent mobility of older adults in Pakistan. They could often participate in sociocultural activities, resulting in reduced social exclusion.

As stated earlier, existing studies [89,97] have noted that the neighborhood social features are related to walking behavior and public transport use. In line with the earlier findings that show significant positive relationships between neighborhood social aspects (such as safety, social cohesion, social interaction, etc.) and activity participation [92,96], this study confirmed that neighborhood social constraints positively influence social exclusion. In other words, older adults who perceive greater social constraints in their neighborhood would prefer not to walk and access public transport, which might restrict their mobility and everyday activities, thus leading to social exclusion.

Similarly, according to our expectations, the relationship between intentions to use public transportation and social exclusion established a significant negative correlation. The findings may be linked to [102,103], who concluded that the greater intention and motivation to accomplish a particular target leads to social participation. The result showed that older adults with intentions to use public transportation could have a strong propensity to be independent and mobile. Hence, frequent traveling with public transport modes might enhance social interactions, and older adults could easily participate in their daily socioeconomic activities. In fact, the process could reduce the feelings of social exclusion among older adults.

### 5.1. Implications

Older adults in Pakistan also face severe mobility issues and social exclusion due to the accessibility issues in the public transport environment [129]. Based on the Integrated Behavioral Model, we demonstrated that the psychosocial aspects such as perceived norms, attitude, and behavioral control beliefs are important factors of public transport use. These results indicated that the efforts to reduce social exclusion can be constrained without accounting for the psychosocial factors of public transport users and have some vital implications for promoting social sustainability in Pakistan.

First, perceived injunctive norms influence the intention to use public transportation, which subsequently affects social exclusion. Hence, soft policies such as awareness campaigns and educational programs should be followed to promote the benefits of public transit or awareness of negative consequences of private mode such as pollution, traffic congestion, greenhouse effect, etc. The education of peers of older adults is incredibly important, which could help realize the potential consequences. The awareness of the social implications of neglecting public transportation should also be provided to peers and older adults. As our findings indicate, the internalization process in which a person embraces a set of values and norms defined by others via social interaction is necessary to encourage the use of public transportation.

Second, as our research indicates that perceived control and personal attitudes significantly predicted the intention to use public transport, awareness programs can also provide older adults with enough knowledge and direct them to develop constructive public transportation attitudes. These educational campaigns might not be as successful as one would anticipate, because the older adults’ shift to public transportation is difficult owing to the constraints in the physical environment, i.e., the need to consider the personal efficacy with the accessibility of public transportation. Realizing the poor digital literacy with using metro services, especially the familiarity with use of automatic fare collection system and the smart cards, the educational campaigns should also be arranged by PMA to improve the efficacy of older adults. The supporting staff at the metro stations could also help and train the older adults in the ticketing mechanism. Similarly, the neighborhood social environment constraints should also be considered to ensure the smooth walkability to public transit.

Hence, educational campaigns should be paired with regulatory actions. In this regard, demand-responsive mobility can partially solve the critical issues encountered by older adults in using public transport (e.g., the presence of ad hoc stops, different onboard services, use of smaller and more comfortable transport means). Similarly, providing age-friendly walking infrastructure, private car-discouraging mechanisms, etc., might also prove useful to encourage the older adults toward the public transport modes and address social exclusion problems. Moreover, age-sensitive policies directing top–down compulsory initiatives and increasing bottom–up voluntary selection of public transport modes are critical to promoting activity participation and reducing the social exclusion among older adults in Pakistan.

### 5.2. Limitations and Future Research

Despite some significant practical and theoretical implications of this research, constraints must be recognized. The survey respondents were the older adults (aged 60 years and above) who only belonged to six neighborhoods of Lahore. A comprehensive study involving diverse population groups and city-wise comparison could help better understand this vital issue. Secondly, it was a cross-sectional study, and future research can longitudinally reproduce and expand these findings. Moreover, this study primarily considered the indirect effect of psychosocial factors on social exclusion through the intention to use public transportation. However, the direct associations between these psychosocial aspects of older adults and social exclusion might offer more interesting insights.

Perceived accessibility is an important aspect that has recently gained attention in public transportation research. Future studies should focus on how the psychosocial factors of public transport users affect the perceived accessibility. Given the ongoing COVID pandemic, future researchers can extend the findings of this research and might consider other psychosocial barriers of public transport users and examine the problem of social exclusion in other developing countries of similar characteristics.

## 6. Conclusions

The phenomenon of social exclusion is crucial for older adults’ health, which is closely linked with the independent mobility and accessibility of public transportation. The global COVID-19 pandemic and the related public transport mobility restrictions have further contributed to this problem. Hence, it is essential to reduce the mobility barriers and social exclusion to ensure a healthy aging society. The overall goal of this research was to highlight the critical psychosocial barriers of public transportation responsible for the social exclusion of older adults. Cross-sectional data were collected from a sample of older adults living in different neighborhoods of Lahore metropolitan to test the proposed hypotheses and model. The findings illustrated that four variables (i.e., perceived injunctive norms, attitudes, self-efficacy, and perceived behavior control) significantly positively affected older adults’ intention to use public transportation, which subsequently influenced social exclusion. Moreover, the direct effect of neighborhood social constraints and public transport habits also influenced social exclusion. This research highlights the significance of the psychosocial aspects of public transport users to examine social exclusion behavior. The findings provide some practical implications for transport planners and public health researchers to promote social sustainability in cities.

## Figures and Tables

**Table 1:** Summary of the measurement model.

Constructs and Items	Loadings	*t*-Value
(EA)—Experiential Attitude (α = 0.868; CR = 0.909; AVE = 0.714)		
Public transport travel is good	0.819	16.558
Public transport travel is useful	0.824	11.135
Public transport travel is responsible	0.897	19.089
Public transport travel is sensible	0.839	17.226
(IA)—Instrumental Attitude (α = 0.777; CR = 0.869; AVE = 0.688)		
Public transport travel reduces carbon dioxide and PM2.5 emissions	0.850	14.86
Public transport travel alleviates energy shortage issues	0.828	13.616
Public transport travel saves money	0.810	13.508
(IN)—Injunctive Norms (α = 0.869; CR = 0.92; AVE = 0.793)		
My closest friends accept me to use public transport	0.922	23.907
My family/partner accept me to use public transport	0.899	20.957
My work colleagues accept me to use public transport	0.848	15.94
(DN)—Descriptive Norms (α = 0.899; CR = 0.937; AVE = 0.831)		
My closest friends use public transport	0.909	23.82
My family/partner use public transport	0.921	25.304
My work colleagues use public transport	0.905	21.062
(PBC)—Perceived Behavioral Control (α = 0.823; CR = 0.892; AVE = 0.734)		
Easy and understandable payment method	0.849	11.931
Easy to obtain public transport services	0.856	14.055
Overall, easy to use public transport services	0.865	16.49
(SEF)—Self-Efficacy (α = 0.961; CR = 0.966; AVE = 0.683)		
Confidence to use public transport if you are feeling tired	0.837	15.803
Confidence to use public transport if you are in a depressive mood	0.839	9.46
Confidence to use public transport if you have lack of motivation	0.831	10.957
Confidence to use public transport if you are in fatigue	0.819	10.848
Confidence to use public transport if you have lack of energy	0.817	10.965
Confidence to use public transport if you are not feeling very well	0.834	9.634
Confidence to use public transport if you have lack of will power, stressed	0.843	12.558
Confidence to use public transport if the weather is bad	0.83	12.901
Confidence to use public transport if you have less time	0.877	15.259
Confidence to use public transport if you have less money	0.838	12.056
Confidence to use public transport if you have lack of social support	0.834	11.178
Confidence to use public transport during early in the morning	0.802	11.36
Confidence to use public transport during nighttime	0.74	9.042
(PTH)—Public Transport Habits (α = 0.844; CR = 0.906; AVE = 0.762)		
Taking public transport has become part of my life	0.879	14.744
It is hard to give up taking public transport	0.907	20.865
I often spontaneously take public transport	0.832	12.92
(NSEC)—Neighborhood Social Environment Constraints (α = 0.921; CR = 0.940; AVE = 0.759)		
You can meet bad people in this neighborhood	0.851	14.877
Acts of vandalism happen in this neighborhood	0.882	21.078
Here in the night there is the risk of dangerous encounters	0.849	15.087
People do not gossip too much in this neighborhood	0.871	19.053
In this neighborhood you feel watched	0.724	12.548
In this neighborhood people are intrusive	0.784	17.146
In this neighborhood it is difficult to make friends with people	0.832	16.958
In this neighborhood it is difficult to get to know people	0.714	15.025
In this neighborhood people tend to be isolated	0.971	19.487
(INT)—Intention to Use Public Transport (α = 0.729; CR = 0.879; AVE = 0.784)		
My intention to use public transportation instead of the private vehicle for my frequent trips in the next seven days is strong.	0.916	22.08
I intend to use public transportation instead of the private vehicle for my frequent trips in the next seven days.	0.854	22.613
(SE)—Social Exclusion (α = 0.762; CR = 0.904; AVE = 0.856)		
Gross household income	0.591 *	0.114
Unemployment status	0.748	0.005
Recent participation in political or community groups	0.749	7.186
Exclusion form range of activities such as hobbies, shopping, leisure etc.	0.867	16.567
Easy to could get help from others if they needed it	0.846	17.296

Notes: AVE = Average variance extracted; CR = Composite reliability; α = Cronbach’s alpha. * indicated items with low factor loadings.

**Table 2:** Fornell–Lacker criterion and Heterotrait–Monotrait (HTMT) ratio values.

Construct	1	2	3	4	5	6	7	8	9	10
DN	0.912	0.812	0.789	0.649	0.72	0.76	0.682	0.73	0.872	0.849
2. NSEC	0.744 **	0.871	0.724	0.463	0.818	0.512	0.537	0.8	0.73	0.796
3. EA	0.781 **	0.828 **	0.845	0.499	0.615	0.641	0.676	0.851	0.559	0.853
4. PTH	0.568 **	0.417 **	0.452 **	0.873	0.684	0.719	0.676	0.795	0.495	0.644
5. IN	0.872 **	0.727 **	0.797 **	0.586 **	0.89	0.589	0.753	0.741	0.809	0.825
6. IA	0.803 **	0.839 **	0.837 **	0.617 **	0.809 **	0.829	0.784	0.749	0.785	0.605
7. INT	0.565 **	0.451 **	0.563 **	0.768 **	0.612 **	0.621 **	0.885	0.695	0.593	0.729
8. PBC	0.787 **	0.696 **	0.722 **	0.678**	0.787 **	0.810 **	0.568 **	0.857	0.85	0.811
9. SEF	0.817 **	0.864 **	0.838 **	0.458 **	0.79 **	0.815 **	0.517 **	0.757 **	0.827	0.825
10. SE	0.665 **	0.636 **	0.663 **	−0.471 **	0.626 **	0.663 **	−0.519 **	0.635 **	0.681 **	0.925

Notes: Fornell–Lacker’s criteria = Italic values on the diagonal are the square root of AVE; values above the diagonal are HTMT values, and below is the correlation between constructs; ** Correlation is significant at the 0.01 level (2-tailed).

**Table 3:** Summary of the result of hypotheses.

Hypothesis	Path	Co-Efficient	*t*-Value	*p*-Value	Conclusion
H1a	EA → Intention	0.569	4.166 ***	0.001	Accepted
H1b	IA → Intention	0.451	2.873 **	0.004	Accepted
H2a	IN → Intention	0.601	8.088 ***	0.001	Accepted
H2b	DN → Intention	0.051	1.345 ^n.s.^	0.321	Rejected
H3a	PBC → Intention	0.121	2.202 *	0.038	Accepted
H3b	SEF → Intention	0.317	2.673 **	0.008	Accepted
H4	PTH → Social Exclusion	−0.193	3.205 ***	0.004	Accepted
H5	NSEC → Social Exclusion	0.496	6.203 ***	0.000	Accepted
H6	Intention → Social Exclusion	−0.226	2.777 **	0.006	Accepted
	*R*^2^_(Intention to use Public Transport)_ = 0.442 *R*^2^_(Social Exclusion (Behavior)_ = 0.469	SRMR composite model = 0.045			

Notes: n.s. = Not significant; * significant with |*t*| ≥ 1.65 at *p* 0.05 level; ** significant with |*t*| ≥ 2.33 at *p* 0.01 level; *** significant with |*t*| ≥ 3.09 at *p* 0.001 level. *R^2^* = Determination coefficients; *R^2^* ≥ 0.25 (weak); ≥ 0.50 (moderate); ≥ 0.75 (substantial); SRMR = Standardized root mean square residual.

## Data Availability

The data presented in this study are available on request from the corresponding authors. The data are not publicly available because certain measures are still need to be used in other studies.

## References

[1] United Nations (2015). World Population Ageing 2015 (ST/ESA/SER. A/390).

[2] DESAU (2017). World Population Prospects, The 2017 Revision, Volume I: Comprehensive Tables.

[3] Walsh K., O’Shea E., Scharf T. (2019). Rural old-age social exclusion: A conceptual framework on mediators of exclusion across the lifecourse. Ageing Soc..

[4] Scharf T., Keating N. (2012). Social exclusion in later life: A global challenge. Exclusion Incl. Old Age.

[5] Colglazier W. (2015). Sustainable development agenda: 2030. Science.

[6] Levin L. (2019). How may public transport influence the practice of everyday life among younger and older people and how may their practices influence public transport?. Soc. Sci..

[7] Kenyon S., Rafferty J., Lyons G. (2003). Social exclusion and transport in the UK: A role for virtual accessibility in the alleviation of mobility-related social exclusion?. J. Soc. Policy.

[8] Lucas K. (2012). Transport and social exclusion: Where are we now?. Transport. Policy.

[9] Levitas R., Pantazis C., Fahmy E., Gordon D., Lloyd-Reichling E., Patsios D. (2007). The Multi-Dimensional Analysis of Social Exclusion.

[10] Delbosc A., Currie G. (2011). The spatial context of transport disadvantage, social exclusion and well-being. J. Transport. Geogr..

[11] De Vos J., Schwanen T., van Acker V., Witlox F. (2013). Travel and subjective well-being: A focus on findings, methods and future research needs. Transport. Rev..

[12] Olsson L.E., Gärling T., Ettema D., Friman M., Fujii S. (2013). Happiness and satisfaction with work commute. Soc. Indic. Res..

[13] He S.Y., Cheung Y.H., Tao S. (2018). Travel mobility and social participation among older people in a transit metropolis: A socio-spatial-temporal perspective. Transp. Res. Part A Policy Pract..

[14] Ignaccolo M., Inturri G., Giuffrida N., Torrisi V. Public Transport Accessibility and Social Exclusion: Making the Connections. Proceedings of the Third International Conference on Traffic and Transport Engineering (ICTTE).

[15] Banister D. (2005). Unsustainable Transport: City Transport in the New Century.

[16] Hui V., Habib K.N. (2014). An Investigation of Transport-Related Social Exclusion of the at-Risk Community (Homeless People) in Toronto, Canada.

[17] Kenyon S. (2011). Transport and social exclusion: Access to higher education in the UK policy context. J. Transport. Geogr..

[18] Mrak I., Campisi T., Tesoriere G., Canale A., Cindrić M. (2019). The role of urban and social factors in the accessibility of urban areas for people with motor and visual disabilities. AIP Conference Proceedings.

[19] Lucas K., Stanley J. (2013). Achieving socially sustainable transport in the development context. Anais da 13th World Con-ference on Transport Research.

[20] McDonagh J. (2006). Transport policy instruments and transport-related social exclusion in rural Republic of Ireland. J. Transport. Geogr..

[21] Church A., Frost M., Sullivan K. (2000). Transport and social exclusion in London. Transport. Policy.

[22] Webber S.C., Porter M.M., Menec V.H. (2010). Mobility in older adults: A comprehensive framework. Gerontologist.

[23] Gorman M., Jones S., Turner J. (2019). Older People, Mobility and Transport in Low-and Middle-Income Countries: A Review of the Research. Sustainability.

[24] Campisi T., Akgün N., Ticali D., Tesoriere G. (2020). Exploring public opinion on personal mobility vehicle use: A case study in Palermo, Italy. Sustainability.

[25] Schwanen T., Páez A. (2010). The mobility of older people: An introduction. J. Transport. Geogr..

[26] World Health Organization (2015). World Report on Ageing and Health.

[27] Nikitas A., Avineri E., Parkhurst G. (2011). Older people‘s attitudes to road charging: Are they distinctive and what are the implications for policy?. Transp. Plan. Technol..

[28] Nikitas A., Avineri E., Parkhurst G. (2018). Understanding the public acceptability of road pricing and the roles of older age, social norms, pro-social values and trust for urban policy-making: The case of Bristol. Cities.

[29] Mifsud D., Attard M., Ison S. (2019). An exploratory study of the psychological determinants of mobility of older people in Malta. Res. Transp. Bus. Manag..

[30] Risser R., Sucha M. (2020). Start Walking! How to Boost Sustainable Mode Choice—Psychological Measures to Support a Shift from Individual Car Use to More Sustainable Traffic Modes. Sustainability.

[31] Donald I.J., Cooper S.R., Conchie S.M. (2014). An extended theory of planned behaviour model of the psychological factors affecting commuters’ transport mode use. J. Environ. Psychol..

[32] Van Holle V., van Cauwenberg J., de Bourdeaudhuij I., Deforche B., van de Weghe N., van Dyck D. (2016). Interactions between neighborhood social environment and walkability to explain belgian older adults’ physical activity and sedentary time. Int. J. Environ. Res. Public Health.

[33] Van Cauwenberg J., de Donder L., Clarys P., de Bourdeaudhuij I., Buffel T., de Witte N., Dury S., Verté D., Deforche B. (2014). Relationships between the perceived neighborhood social environment and walking for transportation among older adults. Soc. Sci. Med..

[34] Van Dyck D., Cardon G., Deforche B., Giles-Corti B., Sallis J.F., Owen N., de Bourdeaudhuij I. (2011). Environmental and psychosocial correlates of accelerometer-assessed and self-reported physical activity in Belgian adults. Int. J. Behav. Med..

[35] Panter J.R., Jones A. (2010). Attitudes and the environment as determinants of active travel in adults: What do and don’t we know?. J. Phys. Act. Health.

[36] Levasseur M., Généreux M., Bruneau J.-F., Vanasse A., Chabot É., Beaulac C., Bédard M.-M. (2015). Importance of proximity to resources, social support, transportation and neighborhood security for mobility and social participation in older adults: Results from a scoping study. BMC Public Health.

[37] Fishbein M., von Haeften I., Appleyard J. (2001). The role of theory in developing effective interventions: Implications from Project SAFER. Psychol. Health Med..

[38] Montaño D.E., Kasprzyk D. (2015). Theory of reasoned action, theory of planned behavior, and the integrated behavioral model. Health Behav. Theoryres. Pract..

[39] Fishbein M., Ajzen I. (2011). Predicting and Changing Behavior: The Reasoned Action Approach.

[40] Gold G.J. (2011). Review of Predicting and Changing Behavior: The Reasoned Action Approach. J.Soc. Psychol..

[41] Zhao S., Li L., Dong Z., Wu B. (2011). Analyzing public transportation use behavior based on the theory of planned behavior: To what extent does attitude explain the behavior?. ICCTP 2011: Towards Sustainable Transportation Systems.

[42] Galdames C., Tudela A., Carrasco J.-A. (2011). Exploring the role of psychological factors in mode choice models by a latent variables approach. Transp. Res. Rec..

[43] Boudreau F., Godin G. (2014). Participation in regular leisure-time physical activity among individuals with type 2 diabetes not meeting Canadian guidelines: The influence of intention, perceived behavioral control, and moral norm. Int. J. Behav. Med..

[44] Wan C., Shen G.Q., Yu A. (2014). The role of perceived effectiveness of policy measures in predicting recycling behaviour in Hong Kong. Resour. Conserv. Recycl..

[45] Schade J., Schlag B. (2003). Acceptability of urban transport pricing strategies. Transp. Res. Part F Traffic Psychol. Behav..

[46] Schade J., Baum M. (2007). Reactance or acceptance? Reactions towards the introduction of road pricing. Transp. Res. Part A Policy Pract..

[47] Al-Rashid M.A., Nahiduzzaman K.M., Ahmed S., Campisi T., Akgün N. (2020). Gender-Responsive Public Transportation in the Dammam Metropolitan Region, Saudi Arabia. Sustainability.

[48] Maramba P., Bamberger M. (2001). A gender responsive monitoring and evaluation system for rural travel and transport programs in Africa. SSATP (Sub-Saharan Africa Transport Policy Program, The World Bank and Economic Commission for Africa) Working Paper.

[49] Plazinić B.R., Jović J. (2014). Women and transportation demands in rural Serbia. J. Rural Stud..

[50] Şimşekoğlu Ö., Nordfjærn T., Rundmo T. (2015). The role of attitudes, transport priorities, and car use habit for travel mode use and intentions to use public transportation in an urban Norwegian public. Transport. Policy.

[51] Lo S.H., van Breukelen G.J., Peters G.-J.Y., Kok G. (2016). Commuting travel mode choice among office workers: Comparing an Extended Theory of Planned Behavior model between regions and organizational sectors. Travel Behav. Soc..

[52] Bopp M., Kaczynski A.T., Besenyi G. (2012). Active commuting influences among adults. Prev. Med..

[53] Lee S.J., Lina Kim H. (2018). Roles of perceived behavioral control and self-efficacy to volunteer tourists’ intended participation via theory of planned behavior. Int. J. Tour. Res..

[54] Faber K., van Lierop D. (2020). How will older adults use automated vehicles? Assessing the role of AVs in overcoming perceived mobility barriers. Transp. Res. Part A Policy Pract..

[55] Kubiszewski V., Auzoult L., Potard C., Lheureux F. (2019). Witnessing school bullying: To react or not to react? An insight into perceived social norms regulating self-predicted defending and passive behaviours. Educ. Psychol..

[56] Ajzen I. (2005). Attitudes, Personality, and Behavior.

[57] Cialdini R.B. (2012). Handbook of Theories of Social Psychology.

[58] Doran R., Larsen S. (2016). The relative importance of social and personal norms in explaining intentions to choose eco-friendly travel options. Int. J. Tour. Res..

[59] Chowdhury S., Zhai K., Khan A. (2016). The Effects of Access and Accessibility on Public Transport Users’ Attitudes. J. Public Transp..

[60] Bamberg S., Hunecke M., Blöbaum A. (2007). Social context, personal norms and the use of public transportation: Two field studies. J. Environ. Psychol..

[61] Kroesen M. (2015). Do partners influence each other’s travel patterns? A new approach to study the role of social norms. Transp. Res. Part. A Policy Pract..

[62] Bourke M., Craike M., Hilland T.A. (2019). Moderating effect of gender on the associations of perceived attributes of the neighbourhood environment and social norms on transport cycling behaviours. J. Transport. Health.

[63] Ingvardson J.B., Nielsen O.A. (2019). The relationship between norms, satisfaction and public transport use: A comparison across six European cities using structural equation modelling. Transp. Res. Part. A Policy Pract..

[64] Zhang D., Schmöcker J.-D., Fujii S., Yang X. (2016). Social norms and public transport usage: Empirical study from Shanghai. Transportation.

[65] De Leeuw A., Valois P., Ajzen I., Schmidt P. (2015). Using the theory of planned behavior to identify key beliefs underlying pro-environmental behavior in high-school students: Implications for educational interventions. J. Environ. Psychol..

[66] Soerjoatmodjo G.W.L. Subjective norms of the intention to use green sustainable transportation: A case study of In-Trans shuttle bus facility and travel mode choice of Pembangunan Jaya University students in Bintaro Jaya. Proceedings of the Applied Psychology: 2015 Asian Congress of Applied Psychology (ACAP 2015).

[67] Ru X., Wang S., Chen Q., Yan S. (2018). Exploring the interaction effects of norms and attitudes on green travel intention: An empirical study in eastern China. J. Clean. Prod..

[68] Simons D., de Bourdeaudhuij I., Clarys P., de Cocker K., de Geus B., Vandelanotte C., van Cauwenberg J., Deforche B. (2017). Psychosocial and environmental correlates of active and passive transport behaviors in college educated and non-college educated working young adults. PLoS ONE.

[69] Armitage C.J., Conner M. (1999). Predictive validity of the theory of planned behaviour: The role of questionnaire format and social desirability. J. Community Appl. Soc. Psychol..

[70] Norman P., Hoyle S. (2004). The theory of planned behavior and breast self-examination: Distinguishing between perceived control and self-efficacy. J. Appl. Soc. Psychol..

[71] Greenslade J.H., White K.M. (2005). The prediction of above-average participation in volunteerism: A test of the theory of planned behavior and the volunteers functions inventory in older Australian adults. J. Soc. Psychol..

[72] McGehee N.G. (2002). Alternative tourism and social movements. Ann. Tour. Res..

[73] Maslow A. (1993). The Farthest Reaches of Human Nature.

[74] McGehee N.G. (2012). Oppression, emancipation, and volunteer tourism: Research propositions. Ann. Tour. Res..

[75] Maddison R., Vander Hoorn S., Jiang Y., Mhurchu C.N., Exeter D., Dorey E., Bullen C., Utter J., Schaaf D., Turley M. (2009). The environment and physical activity: The influence of psychosocial, perceived and built environmental factors. Int. J. Behav. Nutr. Phys. Act..

[76] Thorsen L., Courneya K.S., Stevinson C., Fosså S.D. (2008). A systematic review of physical activity in prostate cancer survivors: Outcomes, prevalence, and determinants. Supportive Care Cancer.

[77] Zoogah D.B. (2010). Why should I be left behind? Employees’ perceived relative deprivation and participation in development activities. J. Appl. Psychol..

[78] Wood W., Rünger D. (2016). Psychology of habit. Annu. Rev. Psychol..

[79] Gardner B. (2015). A review and analysis of the use of ‘habit’in understanding, predicting and influencing health-related behaviour. Health Psychol. Rev..

[80] Gärling T., Axhausen K.W. (2003). Introduction: Habitual travel choice. Transportation.

[81] Verplanken B., Aarts H., van Knippenberg A. (1997). Habit, information acquisition, and the process of making travel mode choices. Eur. J. Soc. Psychol..

[82] Gardner B. (2009). Modelling motivation and habit in stable travel mode contexts. Transp. Res. Part. F Traffic Psychol. Behav..

[83] Verplanken B., Aarts H. (1999). Habit, attitude, and planned behaviour: Is habit an empty construct or an interesting case of goal-directed automaticity?. Eur. Rev. Soc. Psychol..

[84] Beltran J., Wilson O.W., Papalia Z., Duffey M., Bopp M. (2019). Examining the Relationship of Active Transport Habits with Physical Activity Levels in a Diverse Sample of College Students. Ann. Behav. Med..

[85] Sthapit E., Kozak M., Coudounaris D. (2019). What am I going to do now? Examining choice overload in vacation activities using the familiarity concept. Scand. J. Hosp. Tour..

[86] Buys L., Snow S., van Megen K., Miller E. (2012). Transportation behaviours of older adults: An investigation into car dependency in urban Australia. Australas. J. Ageing.

[87] Currie G., Delbosc A. (2010). Exploring public transport usage trends in an ageing population. Transportation.

[88] Rosenbloom S. (2001). Sustainability and automobility among the elderly: An international assessment. Transportation.

[89] Ferreira I.A., Johansson M., Sternudd C., Fornara F. (2016). Transport walking in urban neighbourhoods—Impact of perceived neighbourhood qualities and emotional relationship. Landsc. Urban. Plan..

[90] Amerigo M. (2002). A psychological approach to the study of residential satisfaction. Residential Environments; Choice, Satisfaction, and Behavior.

[91] Fornara F., Bonaiuto M., Bonnes M. (2010). Cross-validation of abbreviated perceived residential environment quality (PREQ) and neighborhood attachment (NA) indicators. Environ. Behav..

[92] Kaczynski A.T., Glover T.D. (2012). Talking the talk, walking the walk: Examining the effect of neighbourhood walkability and social connectedness on physical activity. J. Public Health.

[93] Datar A., Nicosia N., Shier V. (2013). Parent perceptions of neighborhood safety and children’s physical activity, sedentary behavior, and obesity: Evidence from a national longitudinal study. Am. J. Epidemiol..

[94] McDonald N.C. (2007). Travel and the social environment: Evidence from Alameda County, California. Transp. Res. Part D Transp. Environ..

[95] Molnar B.E., Gortmaker S.L., Bull F.C., Buka S.L. (2004). Unsafe to play? Neighborhood disorder and lack of safety predict reduced physical activity among urban children and adolescents. Am. J. Health Promot..

[96] Janssen I. (2014). Crime and perceptions of safety in the home neighborhood are independently associated with physical activity among 11–15 year olds. Prev. Med..

[97] Ma L., Kent J.L., Mulley C. (2018). Transport disadvantage, social exclusion, and subjective well-being. J. Transp. Land Use.

[98] Ajzen I. (1991). The theory of planned behavior. Organ. Behav. Hum. Decis. Process..

[99] Renneberg B., Stäbler K., Fiedler P., Röpke S. (2008). Facial emotional expressions and behavioral intentions as reactions to social exclusion in borderline personality disorder. Int. J. Psychol..

[100] Pfundmair M., Graupmann V., Frey D., Aydin N. (2015). The different behavioral intentions of collectivists and individualists in response to social exclusion. Personal. Soc. Psychol. Bull..

[101] Bacon A. (2017). Effects of Social Exclusion and Familiar others on Alcohol Use Intentions at A Hypothetical House Party. Alcohol. Clin. Exp. Res..

[102] Sano N., Kyougoku M. (2016). An analysis of structural relationship among achievement motive on social participation, purpose in life, and role expectations among community dwelling elderly attending day services. PeerJ.

[103] Alnahdi G.H., Schwab S. (2020). Psychometric Properties of the Arabic Version of the Behavioral Intention to Interact With Peers With Intellectual Disability Scale. Front. Psychol..

[104] Al-Rashid M.A., Rao M.N., Ahmad Z. (2020). Using GIS measures to analyze the spatial equity to public parks in Lahore metropolitan. J. Res. Archit. Plan..

[105] Aziz A., Nawaz M., Nadeem M., Afzal L. (2018). Examining suitability of the integrated public transport system: A case study of Lahore. Transp. Res. Part. A Policy Pract..

[106] Lahore Transport Company, Government of the Punjab Free Transport Card. https://ltc.gop.pk/free_transport_card.

[107] Punjab Masstransit Authority, Government of the Punjab Fare Policy. https://pma.punjab.gov.pk/farepolicy.

[108] Javid M.A., Abdullah S., Hashmi A.I., Akbar M.U., Ghazanfar-Ullah M. (2018). Passengers’ attitudes and preference towards metro-bus service in Lahore. J. Urban. Environ. Eng..

[109] Punjab Masstransit Authority, Government of the Punjab How to Use Our Services. https://pma.punjab.gov.pk/faremedia.

[110] Malik B.Z., ur Rehman Z., Khan A.H., Akram W. (2020). Women’s mobility via bus rapid transit: Experiential patterns and challenges in Lahore. J. Transport. Health.

[111] Ahmad Z., Batool Z., Starkey P. (2019). Understanding mobility characteristics and needs of older persons in urban Pakistan with respect to use of public transport and self-driving. J. Transp. Geogr..

[112] Memon M.A., Ting H., Cheah J.-H., Thurasamy R., Cham F.C.a.T.H. (2020). Sample Size for Survey Research: Review and Recommendations. J. Appl. Struct. Equ. Modeling.

[113] Wan C., Shen G.Q., Choi S. (2017). Experiential and instrumental attitudes: Interaction effect of attitude and subjective norm on recycling intention. J. Environ. Psychol..

[114] Fornara F., Pattitoni P., Mura M., Strazzera E. (2016). Predicting intention to improve household energy efficiency: The role of value-belief-norm theory, normative and informational influence, and specific attitude. J. Environ. Psychol..

[115] Forward S.E. (2014). Exploring people’s willingness to bike using a combination of the theory of planned behavioural and the transtheoretical model. Eur. Rev. Appl. Psychol..

[116] Chen C.-F., Chao W.-H. (2011). Habitual or reasoned? Using the theory of planned behavior, technology acceptance model, and habit to examine switching intentions toward public transit. Transp. Res. Part F Traffic Psychol. Behav..

[117] de Geus B., Wuytens N., Deliens T., Keserü I., Macharis C., Meeusen R. (2019). Psychosocial and environmental correlates of cycling for transportation in Brussels. Transp. Res. Part. A Policy Pract..

[118] Chen W., Cao C., Fang X., Kang Z. (2019). Expanding the Theory of Planned Behaviour to Reveal Urban Residents’ Pro-Environment Travel Behaviour. Atmosphere.

[119] Klöckner C.A., Blöbaum A. (2010). A comprehensive action determination model: Toward a broader understanding of ecological behaviour using the example of travel mode choice. J. Environ. Psychol..

[120] Currie G., Delbosc A. (2010). Modelling the social and psychological impacts of transport disadvantage. Transportation.

[121] Henseler J., Ringle C.M., Sarstedt M. (2015). A new criterion for assessing discriminant validity in variance-based structural equation modeling. J. Acad. Mark. Sci..

[122] Anderson J.C., Gerbing D.W. (1982). Some methods for respecifying measurement models to obtain unidimensional constructs measures. J. Mark. Res..

[123] Chin W.W. (1998). Issues and Opinion on Structural Equation Modeling. Mis Q..

[124] Hair J.F., Hult G.T.M., Ringle C., Sarstedt M. (2016). A Primer on Partial least Squares Structural Equation Modeling (PLS-SEM).

[125] Fornell C., Larcker D.F. (1981). Structural Equation Models with Unobservable Variables and Measurement Error: Algebra and Statistics. J. Mark. Res..

[126] Hulland J. (1999). Use of Partial Least Squares (PLS) in Strategic Management Research: A Review of Four Recent Studies. Strateg. Manag. J..

[127] Cohen J. (1992). Statistical Power Analysis. Curr. Dir. Psychol. Sci..

[128] Henseler J., Dijkstra T.K., Sarstedt M., Ringle C.M., Diamantopoulos A., Straub D.W., David J., Ketchen J., Hair J.F., Hult G.T.M. (2014). Common Beliefs and Reality about PLS. Organ. Res. Methods.

[129] Adeel M. (2015). Transportation Disadvantage and Social Exclusion in Pakistan.

